# AGE-RAGE signal generates a specific NF-κB RelA “barcode” that directs collagen I expression

**DOI:** 10.1038/srep18822

**Published:** 2016-01-05

**Authors:** Yunqian Peng, Ji-Min Kim, Hal-Sol Park, Annie Yang, Celia Islam, Edward G. Lakatta, Li Lin

**Affiliations:** 1 Laboratory of Cardiovascular Sciences, National Institute on Aging, National Institute of Health, Baltimore, MD 21224.

## Abstract

Advanced glycation end products (AGEs) are sugar-modified biomolecules that accumulate in the body with advancing age, and are implicated in the development of multiple age-associated structural and functional abnormities and diseases. It has been well documented that AGEs signal via their receptor RAGE to activate several cellular programs including NF-κB, leading to inflammation. A large number of stimuli can activate NF-κB; yet different stimuli, or the same stimulus for NF-κB in different cellular settings, produce a very different transcriptional landscape and physiological outcome. The NF-κB barcode hypothesis posits that cellular network dynamics generate signal-specific post-translational modifications, or a “barcode” to NF-κB, and that a signature “barcode” mediates a specific gene expression pattern. In the current study, we established that AGE-RAGE signaling results in NF-κB activation that directs collagen Ia1 and Ia2 expression. We further demonstrated that AGE-RAGE signal induces phosphorylation of RelA at three specific residues, T254, S311, and S536. These modifications are required for transcription of collagen I genes and are a consequence of cellular network dynamics. The increase of collagen content is a hallmark of arterial aging, and our work provides a potential mechanistic link between RAGE signaling, NF-κB activation, and aging-associated arterial alterations in structure and function.

Advanced glycation end products (AGEs) are sugar cross-linked biomolecules generated by non-enzymatic Maillard reaction *in vivo* and *in vitro*^1^. AGEs are formed within the body; they can also be ingested from food. These heterogeneous macromolecules accumulate in the body with advancing age of humans and animals that affect tissue properties and promote inflammation^2^. In the context of hyperglycemia and diabetes, the level of AGEs production that accompanies advancing age is accelerated, further enhancing tissue damages and inflammation^3^. The receptor for advanced glycation end products (RAGE) is the main receptor for AGEs and a focal point to transmit inflammatory signals to cellular programs^4^. Like Toll-like receptors (TLRs), RAGE is a pattern recognition receptor that interacts with multiple, highly diverse ligands. Similar to TLRs, RAGE signaling also activates NF-κB, a master regulator of inflammation, and the receptor shares a subgroup of ligands with TLRs, including high mobility group box-1(HMGB1), s100 family members, and lipopolysaccharide (LPS)^5^,^6^,^7^. Recently, putative cellular factors that relay RAGE signal to NF-κB have been identified^8^. It remains to be studied whether each RAGE/ligand pair generates a distinct inflammatory profile, and if so, through what mechanism.

NF-κB is a heterodimeric (RelA/p50) transcription factor that binds to the consensus motif in the promotor and/or enhancer in the regulatory region of a target gene. Binding of NF-κB to the regulatory *cis* elements enables its interactions with other transcriptional factors and hence influences the transcription of target genes^9^. NF-κB activation is subject to highly complex, multi-layer of regulation resulting from signaling network cross-talks. Therefore, upon different stimuli and physiological contexts, NF-κB activation can result in very different gene expression, and perhaps inflammatory, profiles. RelA (also termed p65) of the prototypical NF-κB heterodimer contains a transcription activation domain, and is often the driver for transcription activation. RelA can be post-translationally modified at both Rel homology DNA binding domain, as well as the tans-activating domain^10^,^11^. Different modifications of RelA may alter its accessibility to a promoter/enhancer, its interactions with its inhibitor IκB and other cellular factors, and its cellular stability and localization, thus influencing its transcriptional targets as well as the transcriptional dynamics^12^. The NF-κB barcode theory hypothesizes that stimuli, via specific cellular signaling networks, generate a unique post-translational modification (PTM) pattern (*i.e.* “barcode”) on NF-κB, and such signature “barcode” drives transcription of a specific group of genes^12^,^13^,^14^. Examples supporting this hypothesis include human pathogen nontypeable *Haemophilus influenzae* (NTHi) and transforming growth factor-β(TGF-β) co-induction generated RelA K221 acetylation and S276/S536 phosphorylation that directs cellular inflammatory responses against NTHi infection^15^; and tumor necrosis factor-α (TNF-α)-induced RelA S468 and S536 phosphorylation that mediates a subset of downstream gene expression^14^. Decoding of “barcodes” or combination of PTMs of NF-κB in respective to an age- or disease-associated stimulus may give rise to the development of precision drugs that target a stereotypically modified NF-κB, and perhaps render reduction of cytotoxicity due to global inhibition of this transcription factor.

Arterial aging is manifested through maladaptation of the vessel wall to various environmental assaults during the lifetime^16^. Hallmarks of such remodeling include a significant increase of collagen contents, crosslinking of proteins on the vessel wall, and the alteration of the composition of the arterial extracellular matrix^17^,^18^. It has been demonstrated that RAGE expression in the vessel is upregulated with aging; and that arterial injury and diabetic condition enhance RAGE expression^19^,^20^. Collagen I and III are the two major collagen species expressed in the arterial wall^21^. A recent work has shown that, compared to wild-type mice, the expression of collagen I and III is significantly decreased in the injured artery of RAGE-null mice, suggesting that RAGE signaling may play a role in collagen I and III production^22^. A collagen I protein consists of 1a1 and 1a2 chains, and computational analysis of regulatory sequence shows that murine *col1a1* and *col1a2* genes both contain a long 5′ regulatory sequence, which encompasses multiple putative NF-κB binding motifs, whereas the regulatory sequence of the *col3a1* gene does not contain any of NF-κB binding *cis* elements^23^. Here, we showed that AGEs stimulation upregulates collagen I expression in the cell, and this up-regulation is RAGE-dependent. We further showed that an AGEs-induced NF-κB RelA barcode is required for collagen I expression, and the generation of this barcode requires activation of specific cellular signaling networks. Our studies are the first to demonstrate an AGE-RAGE signal-generated NF-κB RelA barcode for collagen production, and provide a potential mechanistic link between AGE-RAGE signaling and aging- and disease-associated structural and functional changes in the vasculature.

## Results

### AGE-RAGE axis induces collagen I expression via NF-κB

**Figure 1:**
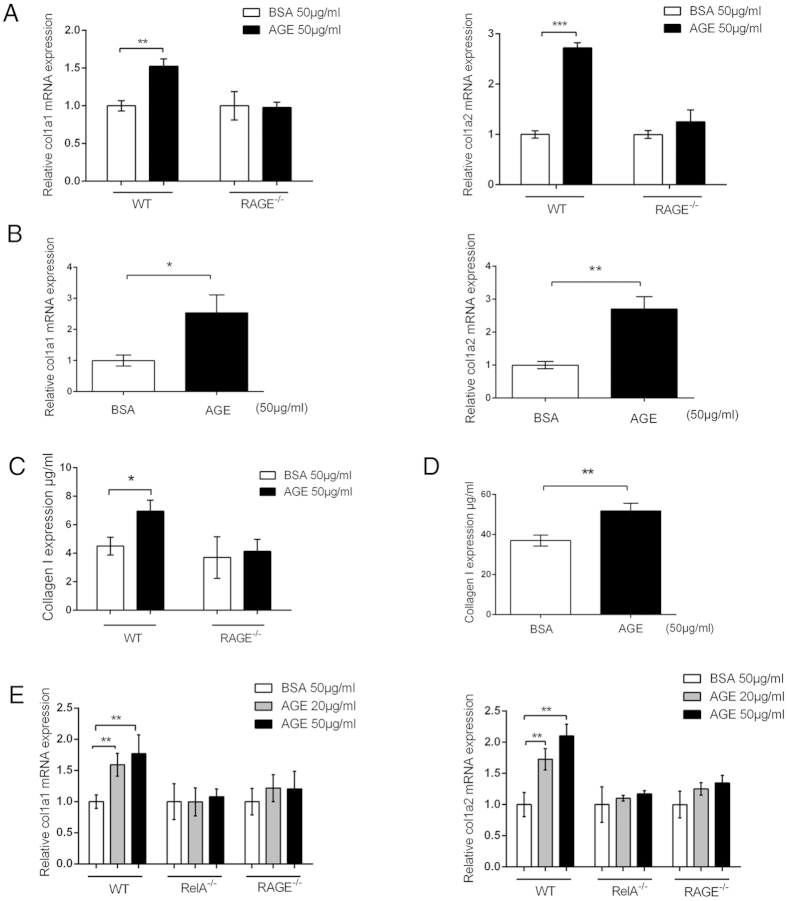
AGE-RAGE axis induces the expression of collagen 1a1 and 1a2 via NF-κB. (**A**) AGEs induce transcription of *col1a1* and *col1a2* genes in murine macrophages. Peritoneal macrophages were isolated from C57BL/6 WT and RAGE-null mice and treated with AGEs for 2 h. Total RNAs were then extracted for qRT-PCR to measure *col1a1* and *col1a2* transcripts. (**B**) AGEs induce transcription of *col1a1* and *col1a2* genes in murine VSMCs. The cells were treated with AGEs for 3 h prior to total RNA extraction and qRT-PCR analyses for *col1a1* and *col1a2* transcripts. (**C,D**), ELISA analysis of collagen I protein in macrophages (**C**) and murine VSMCs (**D**).(**E**) AGE-RAGE signals to NF-κB for *col1a1* and *col1a2* transcription. WT, RAGE-null, and RelA-null MEFs were stimulated with 2 doses of AGEs (20 and 50 μg/ml) for 3 h prior to RNA extraction and qRT-PCR analysis for *col1a1* and *col1a2* transcripts. Data from qRT-PCR and ELISA were from three independent experiments (n = 3), and each sample was measured in triplicate. Readings from cells treated with BSA were used as the negative control. The results were expressed as mean ± SEM. *p < 0.05; **p < 0.01; ***p < 0.001.

We observed that although AGEs accumulate with aging in murine artery wall, collagen I level is lower in the vessel of RAGE-null than that of the wild-type (WT) mice (Fig. S1). To test whether AGEs induce collagen I expression, we first stimulated macrophages isolated from WT and RAGE-null mice with AGEs and measured collagen I expression with real-time quantitative reverse transcription polymerase chain reaction (qRT-PCR), using primers specific to murine *col1a1* and *col1a2* genes. Both genes respond to AGEs stimulation in WT, but not in RAGE-null macrophages (Fig. 1A). Similar results were obtained from murine vascular smooth muscle cells (VSMC) (Fig. 1B), another cell type known to express RAGE^24^. The overall collagen I production in cell lysates from AGEs-stimulated macrophages and VSMCs also appeared to be enhanced (Fig. 1C,D), suggesting that AGEs can induce collagen I expression. To test whether AGEs signal, via RAGE, to NF-κB, leading to collagen I expression, we used RelA-null murine embryonic fibroblast (MEF) cells, as NIH 3T3 MEFs have been known to express RAGE^25^. While AGEs stimulate transcription of *col1a1* and *col1a2* genes in WT MEFs, RelA-null and RAGE-null MEFs have basal level transcription of the two genes only (Fig. 1E). These observations implicate AGE-RAGE axis-induced NF-κB activity in collagen I production.

**Figure 2:**
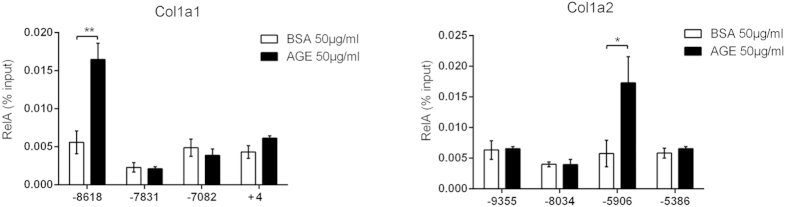
ChIP assays of AGEs-induced RelA binding to 5′ regulatory loci of *col1a1* and *col1a2* genes. MEF cells were treated with either BSA or AGEs for 3 h prior to harvest for ChIP assays. The location of each putative NF-κB binding motif relative to the transcription initiation site was marked underneath each group. All qPCR data were from three independent experiments (n = 3), and each sample was measured in triplicate. The results were expressed as mean ± SEM. *p < 0.05; **p < 0.01.

Although both murine *col1a1* and *col1a2* genes contain a long 5′ upstream regulatory sequence that encompasses several individual, or overlapping, putative NF-κB binding motifs^23^, it is unknown whether these motifs are accessible to NF-κB upon AGEs stimulation. We used chromatin immunoprecipitation (ChIP) analyses to locate the NF-κB binding motifs that are responsive to AGEs stimulation. The 5′ regulatory sequence of *col1a1* and *col1a2* genes both contains four putative NF-κB binding motifs. However, upon AGEs treatment, NF-κB binds to only one putative motif in the 5′ regulatory region of either *col1a1* (–8618) or *col1a2* (–5906) gene (Fig. 2). These results suggest that AGEs stimulation leads to a selective access of NF-κB to the regulatory sequence of collagen I, and that this limited access and binding appears to be sufficient to mediate the transcription of *col1a1* and *colia2* genes.

### AGEs stimulation results in a specific NF-κB barcode that directs collagen I expression

Multiple residues in NF-κB RelA have been found to be post-translationally modified^10^,^11^. Different stimuli or cell settings often result in a different combination of RelA PTMs, which has been coined as stimuli-specific “barcode”^14^. RelA can be modified by both acetylation and phosphorylation; we therefore first tested whether AGEs enhance RelA acetylation, using antibodies specific to acetylated lysine residue. Western blotting of immunoprecipitated RelA from AGEs–treated MEFs showed no enhancement of acetylation except that of basal level, compared to bovine serum albumin (BSA) –treated negative control, suggesting that AGEs do not specifically induce this category of modifications in RelA (Fig. S3A). Consistent with this observation, we also found that, upon AGEs induction, transcriptions of *col1a1* and *col1a2* genes were not affected in RelA-null MEFs reconstituted with RelA (K218R/K221R/K310R) composite mutant, which eliminates all three known potential RelA acetylation sites (Fig. S3B).

**Figure 3:**
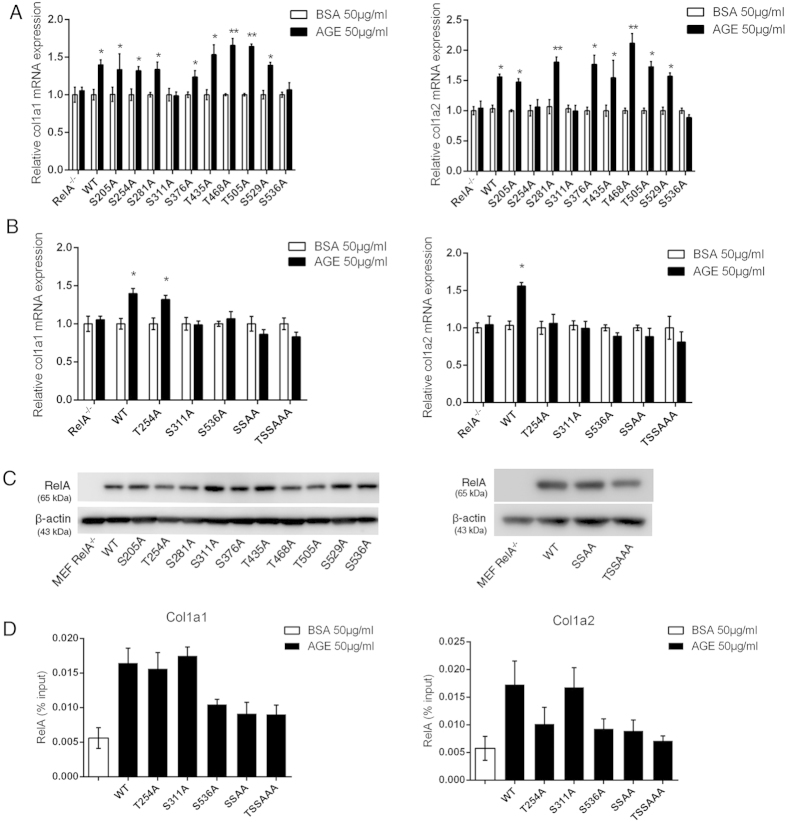
Deciphering AGEs-induced RelA barcode required for transcription of *col1a1* and *col1a2* genes. (**A**) qRT-PCR assessment of AGEs-induced transcription of *col1a1* and *col1a2* genes in RelA mutant-reconstituted MEFs. (**B**) qRT-PCR assessment of AGEs-induced transcription of *col1a1* and *col1a2* genes in reconstituted MEFs harboring RelA(S311A/S536A), (marked as SSAA) and RelA(T254A/S311A/S536A, marked as TSSAAA) composite mutants. (**C**) Western blots using anti-RelA antibodies to assess the expression of RelA/mutants in reconstituted RelA-null MEFs. (**D**) ChIP analyses of the binding of RelA phosphorylation mutants to *col1a1* –8618 locus in reconstituted MEF cells: RelA WT, T254A and S311A AGEs- treated vs. BSA-treated: *p* < 0.01; RelA S536A, SSAA, and TSSAAA AGEs-treated vs. BSA-treated, *p* > 0.05. Among AGEs-treated groups: RelA T254A and S311A vs. WT, *p* > 0.05; RelA S536A, SSAA, and TSSAAA vs. WT, *p* < 0.05. (**E**) ChIP analyses of the binding of RelA phosphorylation mutants to *col1a2* –5906 locus in reconstituted MEF cells: RelA WT and S311A AGEs-treated vs. BSA-treated: *p* < 0.01; RelA T254A, S536A, SSAA and TSSAAA vs. BSA-treated: *p* > 0.05; Among AGEs-treated groups: RelA S311A vs. WT, *p* > 0.05; RelA T254A, S536A, and SSAA vs. WT, *p* < 0.05; RelA TSSAAA vs. WT, *p* < 0.01. For both (**D,E**), the data in BSA-treated group shown in the figure include both WT and RelA mutants (n = 18), and data in individual RelA group treated with AGEs were from 3 independent experiments (n = 3) and each sample was measured in triplicate. All results were expressed as mean ± SEM.

RelA contains at least ten known potential phosphorylation sites^11^. Although there are antibodies to a few individual phosphorylation residues of RelA, not all potential phosphorylation residues in RelA have available antibodies that recognize them. To decipher the RelA “barcode” induced by AGEs stimulation, we mutated all ten potential phosphorylation residues individually in RelA from either T or S to A, and transfected RelA-null MEFs with expression vectors carrying cDNA of individual RelA mutant to generate ten cell lines that stably express the RelA mutant proteins. Stimulation of each reconstituted RelA mutant MEF cell line with AGEs followed by qRT-PCR with primers specific to *col1a1* and *col1a2* genes showed that while AGEs-induced phosphorylation of two residues in RelA, S311 and S536, are critical for transcription of *col1a1* gene, transcription of *col1a2* gene requires one additional AGEs-induced phosphorylation on T254 residue (Fig. 3A). The expression of individual RelA mutant in the stable cell line was verified by western blotting with anti-RelA antibodies (Fig. 3C).

In addition to the assessment of single mutations of RelA phosphorylation target residues on collagen I transcription, we also reconstituted RelA-null cells with RelA(S311A/S536A) and RelA(T254A/S311A/S536A) composite mutants. Stimulation of these two MEF cell lines with AGEs showed that, compared to individual mutations, there is no significant difference of the level of transcription reduction of *col1a1*and *col1a2* genes by the composite mutations (Fig. 3B). These results suggest that the AGEs-induced RelA barcode is likely to act as a constrained entity for transcription of collagen I genes.

PTMs of RelA differentially regulate its bioactivities, dynamics, and cellular localization. To test whether AGEs-induced RelA barcode affects NF-κB binding to the 5′ regulatory region of *col1a1* or *col1a2* genes, we performed additional ChIP assays in MEF cells that stably express RelA proteins containing individual and composite T254A, S311A and S536A mutations. As shown in Fig. 3D, while S536A mutation in RelA affects its binding to both *col1a1* (–8618) and *col1a2* (–5906) consensus motifs, S311A mutation does not impact RelA binding to either locus. In addition to S536A, T254A mutation also affects the binding of RelA to *col1a2* (–5906) locus. It also appears that the binding of *col1a2* regulatory locus is affected by RelA mutations more significantly than that of *col1a1* locus. These results are consistent with those from qRT-PCR analyses (Fig. 3A,B), and suggest that the AGEs-induced RelA barcode, at least in part, exerts its function by regulating RelA binding to the regulatory loci of *col1a1* and *col1a2* genes.

### The cellular network dynamics determine AGE-RAGE signal-induced NF-κB barcode for transcription of collagen I genes

**Figure 4:**
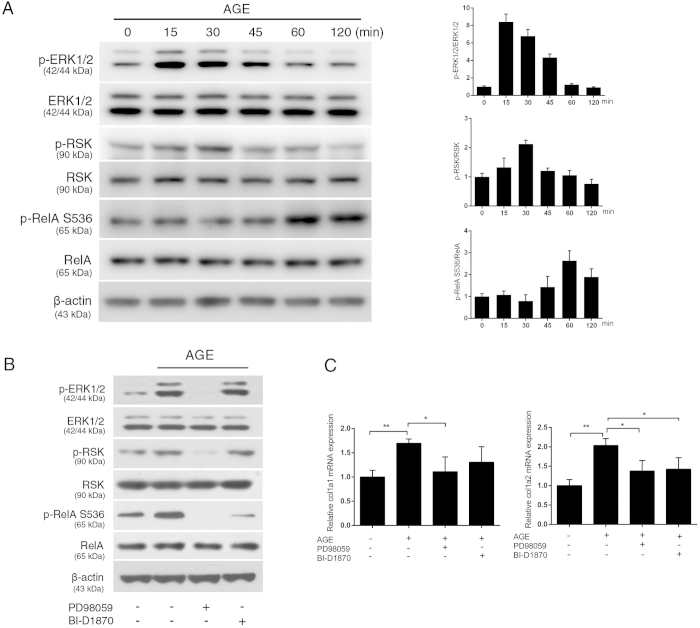
AGEs activate the MAPK/ERK–Rsk-1 pathway to generate a RelA barcode. (**A**) Sequential activation of ERK1/2 and Rsk-1, and phosphorylation of RelA S536 residue. Total cell lysates (10 μg) were analyzed and β-actin was used as overall loading control. Left panels are the representative western blots, and right panels are the densitometric analyses of three western blots. (**B**) Pharmacological inhibition of the MAPK/ERK pathway blocks RelA S536 phosphorylation. MEF cells were pre-treated with ERK inhibitor PD98058 (10 nM), or Rsk-1 inhibitor BI-D1870 (10 μM) for 1 h prior to AGEs stimulation. Western blots were performed using 10 μg of total cell lysates. (**C**) Pharmacological blockers to ERK1/2 and Rsk-1 down-regulate AGEs-induced transcription of *col1a1* and *col1a2* genes in MEF cells. Specific transcripts were analyzed with qRT-PCR (n = 3). The results were expressed as mean ± SEM. *p < 0.05; **p < 0.01.

Post-translational modifications of NF-κB, as well as the transcription of its target genes are the consequence of the cellular networks in response to a specific stimulus. To study the impact of cellular network signaling on AGEs-induced NF-κB barcode and on collagen I expression, we tested whether pharmacologically blocking candidate kinases that target specific RelA residues would also affect collagen I expression. Multiple stimuli can result in phosphorylation of RelA S536 residue and the candidate kinases have been reported to be the IκB kinases (IKKs), ribosomal s6 kinase-1 (Rsk-1, also termed as p90^rsk^), and cyclin-dependent kinase 6 (CDK6)^26^,^27^,^28^,^29^,^30^. Because RAGE signal rapidly activates extracellular signal-regulated kinase (ERK)^31^,^32^, and because Rsk-1 is a direct target of ERK1/2^33^, we tested whether pharmacologically blocking of MAPK/ERK pathway components ERK1/2 and Rsk-1 would down-regulate AGEs-induced transcription of *col1a1* and *col1a2* genes. The activation of ERK1/2 requires signal-induced phosphorylation of its residues T202 and Y204. To verify that AGE-RAGE signal relay leads to phosphorylation of RelA S536, we first assessed the phosphorylation (activation) status of ERK1/2, Rsk-1, and RelA S536 during a time course of AGEs stimulation, using antibodies specific to phosphorylated residues in these proteins. As shown in Fig. 4A, while 15 min treatment of AGEs activates ERK1/2 in MEFs, it takes 30 min to phosphorylate Rsk-1, and at least 60 min for S536 in RelA to be phosphorylated. We further demonstrated that blocking ERK1/2 with an inhibitor, PD98059^34^, blocks AGEs-induced ERK1/2 phosphorylation, and that, in turn, reduces phosphorylation of downstream target Rsk-1 as well as its putative target RelA S536 (Fig. 4B). These results suggest that AGEs indeed induce RelA S536 phosphorylation via ERK1/2 – Rsk-1 signaling pathway. This point is further strengthened by experiments that blocked RelA S536 phosphorylation with an Rsk-1 inhibitor BI-D1870. BI-D1870 does not affect phosphorylation of Rsk-1 by ERK1/2 but inhibits Rsk-1′s catalytic activity^35^. MEFs treated with BI-D1870 and induced with AGEs showed reduced, but not complete abrogation of phosphorylation of RelA S536 (Fig. 4B). As expected, Erk1/2 inhibitor PD98059 significantly down-regulated AGE-induced transcription of *col1a1* and *col1a2* genes (Fig. 4C).While BI-D1870 significantly reduced transcription of *col1a2* gene, the reduction of *col1a1* gene transcription is not statistically significant, which is likely due to the incomplete blocking of Rsk-1by BI-D1870 (Fig. 4B) and a less stringent requirement of the RelA barcode by *col1a1* gene.

It has been reported that protein kinase Cζ (PKCζ) phosphorylates RelA S311^36^, and in addition to the results obtained from reconstituted RelA mutant MEF cell lines (Fig. 3A), we also detected AGEs-induced phosphorylation of RelA S311 (Fig. S4A and C). However, we were unable to block the phosphorylation of RelA S311 using PKCζ pseudosubstrate as an inhibitor^37^(Fig. S4E). These results suggest that PKCζ may not be the kinase that responds to AGEs stimulation and phosphorylates RelA S311. RelA is also subject to signal-induced Pin1 peptidyl-prolyl isomerase-mediated prolyl isomerization, which occludes RelA’s interaction with IκBα and its nuclear export, resulting in a stabilized RelA for transcriptional activities^38^. The prerequisite for RelA prolyl isomerization at P255 is the phosphorylation of the neighboring T254^38^. Although we detected AGEs-induced phosphorylation of RelA T254 (Fig. S4A and B), we could not block AGEs-induced RelA T254 phosphorylation using Pin1 blocker AG-17724 (Fig. S4D). These negative results suggest that AGEs may activate a group of kinases that target RelA S311 and T254, but identities of these kinases are currently unknown.

## Discussion

Arterial aging is manifested through remodeling of the vessel wall, resulting in stiffness and ensuing cardiovascular malaises^16^. The remodeling process involves not only inflammation-induced proliferation of vascular cells, but also alteration of the mural composition including a significant increase of collagen content. Accumulation of AGEs in the body with advancing age has been associated with various pathophysiological consequences such as enhanced arterial stiffness and development of chronic, low-grade inflammation^2^,^39^,^40^,^41^. AGEs stimulation has been known to activate multiple cellular signaling programs including NF-κB, a master regulator of inflammatory phenotypes and cell proliferation^31^,^42^,^43^,^44^,^45^,^46^. However, a direct connection between AGE-RAGE axis and major arterial aging phenotypes including vessel thickening and stiffness had never been established. Our current work provides a clear link between AGE-RAGE signaling and its manifestation on the vasculature by revealing the AGEs signal-induced NF-κB RelA barcode that is required for collagen I production.

The initial clue that RAGE signaling is associated with collagen expression in the vessel came from results of an acute arterial injury model: RAGE-null mice produce a significantly lower level of collagens I and III than that of wild-type mice in the injured vessel^22^. Upon examination of aorta sections from normal, uninjured young (8 – 9 weeks) and old (40–45 weeks) mice of WT and RAGE-null genotypes, we found that, there is a higher level of collagen I in vessels of WT, than that of RAGE-null mice within the same age group. In addition, collagen I expression in vessels is also higher in the older than that of younger mice within the same genotype group (Fig. S1). Similar results have been obtained from rhesus monkey aortic samples (Fig. S2). These observations, together with aging-associated AGEs accumulation throughout the body, suggest that AGE-RAGE signaling axis plays a role in the augmentation of collagen production during arterial aging.

Among the four putative NF-κB binding motifs within the 5′ regulatory region of *col1a1* or *col1a2* gene, only one motif is bound by RelA upon AGEs stimulation (Fig. 2), suggesting that the access of NF-κB transcription factor to the specific regulatory locus of either gene is selective and conditional. The role of NF-κB in regulating collagen I expression has been controversial: a number of previous studies have found that RelA inhibits collagen I expression in murine and human fibroblasts^47^,^48^,^49^,^50^,^51^, whereas other studies found that NF-κB activities are actually required for collagen I expression in various cell types^52^,^53^,^54^,^55^. Our discovery of AGEs-induced unique RelA barcode (Fig. 3) and our observation of the selective access of RelA to the 5′ regulatory locus of *col1a1* and *col1a2* genes (Fig. 2) provide a tangible explanation of the apparent paradoxical role of NF-κB in regulating collagen I expression. Previous works as well as our current studies demonstrate that a specific stimulus generates a characteristic RelA barcode, and that these specific modifications of RelA appear to render its contingent binding to a regulatory locus and dictate its transcriptional dynamics, resulting in a distinctive transcriptional landscape^10^,^11^,^12^,^14^,^15^. Besides the signature RelA barcode, stimuli or cellular signals can also induce a conditional change in the chromosomal structure, rendering the access of transcription factors to specific regulatory loci. Therefore, it is possible that different stimuli or cellular environment generate different RelA barcodes that render its access to a different NF-κB binding motif(s) in the 5′ regulatory region of *col1a1* and *col1a2* genes, leading to inhibition, rather than activation, of collagen I transcription as observed in previous studies.

Both NF-κB binding motifs in collagen I genes that respond to AGEs signal are in a 5′ regulatory locus quite distal to the transcription initiation site (Fig. 2, –8618 for *col1a1*, and –5906 for *col1a2* gene). The long-range of these *cis*-elements relative to their transcription initiation sites has made additional verification using conventional plasmid-, and cell-based reporter assays technically difficult. The involvement of long-range *cis*-elements in transcription regulation has been widely reported, and the roles of long-range *cis*-elements in transcription of target genes have been confirmed using chromosome conformation capture and transgenic reporter analyses in animals^56^. Interestingly, computational and experimental analysis of murine *col1a1* found multiple DNase I -hypersensitive sites and stress-induced DNA duplex destabilization sites in the distal 5′ regulatory region encompassing the NF-κB binding locus; and fragments containing these sites showed strong nuclear matrix binding activity *in vitro*^57^. These circumstantial evidences support the notion that the two distal *cis*-NF-κB binding elements play a regulatory role in collagen I transcription.

Results from RelA mutant-reconstituted MEF cell lines showed that AGEs-induced transcription of *col1a1* gene requires phosphorylation of S311 and S536 in RelA only, whereas transcription of *col1a2* gene requires phosphorylation of one additional residue, T254 (Fig. 3A). PTMs in RelA differentially regulate its bioactivities, transcriptional dynamics, and cellular localizations. Our additional ChIP analyses in RelA mutant cell lines showed that while AGEs-induced phosphorylation at RelA S536 and T254 is required for the binding of NF-κB to the 5′ regulatory loci (S536 for both *col1a1* and *col1a2* genes, and T254 for *col1a2* gene), phosphorylation of RelA S311 appears to act in other regulatory capacity rather than binding to the enhancer (Fig. 3D). These results suggest that AGEs-induced barcode, at least in part, regulates NF-κB binding to the *cis*-regulatory elements of *col1a1* and *col1a2* genes (Fig. 3D). A functional collagen I protein is a triplet fibrillar bundle composed of two collagen Ia1, and one collagen Ia2 chains. The requirement of a relatively “loosened” double code S311/S536 for the transcription of *col1a1* gene verses a relatively “tight” triple code T254/S311/S536 for the transcription of *col1a2* gene may arguably be an advantageous cellular strategy for the biogenesis of a stoichiometrically correct collagen I protein under AGEs stimulation. The different stringency requirement of RelA barcode for the transcription of the *col1a1* and *col1a2* genes is also reflected in the sensitivity of the two genes to pharmacological inhibitors that inhibit upstream kinases in the signaling network: Rsk-1 inhibitor BI-D1870, which only partially blocks RelA S536 phosphorylation, is sufficient to significantly reduce the transcription of the *col1a2* gene, but not that of the *col1a1* gene (Fig. 4C).

Our work also demonstrated that the generation of a specific RelA barcode is the consequence of cellular network dynamics. An AGE-RAGE signal activates the MAPK/ERK, which in turn relays that signal to Rsk-1. The latter phosphorylates RelA S536, completing a part of the common RelA barcode for the transcription of both *col1a1* and *col1a2* genes (Fig. 4). Parallel to this MAPK/ERK signaling route that directly targets RelA, AGE-RAGE signal also activates the IKK signalosome that phosphorylates IκBα for its subsequent proteasome-mediated degradation, releasing the NF-κB complex for nuclear translocation^58^. Apparently, AGEs stimulation induces multiple cellular programs and is likely to generate a transcriptional profile distinctive from that by other NF-κB stimuli, and the enhanced transcription of collagen I genes observed in our studies is only a part of this transcriptional landscape. Our strategy to establish the link between AGE-RAGE signaling and collagen I production has led to the decoding of the first RAGE signal-generated RelA barcode. However, the current work has not yet assessed the overall transcriptional profile generated by AGEs stimulation. Nor is it clear whether T254/S311/S536 is the sole RelA barcode induced by the AGE-RAGE signal. Future work should address these aspects including identification of the responsible pathways leading to AGEs-induced phosphorylation of RelA T254 and S311.

## Methods

### Cells used in the study

RelA-null MEFs were initially generated by Dr. Amer Beg (Moffitt Cancer Center), and together with the wild type MEFs were gifts from Dr. Yosef Anrather (Weil Medical College) with the permission from Dr. Beg. RAGE-null MEFs were isolated from embryos of pregnant RAGE-null mice at 13 days post-coitum, according to the protocol described^59^. MEFs were cultured in Dulbecco’s modified Eagle’s medium (DMEM, Invitrogen) supplemented with 10% fetal bovine serum (FBS, Gibco), 2 mM GlutaMAX (Gibco), and antibiotics. Murine peritoneal macrophages were elicited with peritoneal injection of 1 ml of 3% Brewer thioglycollate medium, and isolated 4 days post-injection according to the protocol described^60^. Macrophages were cultured in DMEM/F12 medium (Invitrogen) supplemented with 10% FBS and antibiotics. Primary murine VSMCs were purchased from Cell Biologics and cultured in gelatin-coated dishes with the medium supplied by the manufacturer. The use of mice and isolation of MEFs and macrophages have been approved by the Animal Care and Use Committee of National Institute on Aging, NIH, and complied with the Guide for the Care and Use of Laboratory Animals (NIH publication no. 3040-2, revised 1999).

### AGEs stimulation and inhibitor treatment

For macrophages, approximately 0.5 × 10^6^ cells were seeded in 35 mm plate and after overnight incubation, cells were incubated in the medium without serum for 20 h prior to AGEs stimulation. AGEs (glycated BSA, BioVision Technologies) were added to cells at indicated final concentration for 2 h prior to harvest for qRT-PCR analysis. BSA was used as the negative control. For VSMCs and MEFs, the cells were seeded and serum-starved similarly as macrophages and AGEs stimulation was 3 h, or as indicated in the individual experiment. ERK1/2 inhibitor PD98059 was purchased from Calbiochem, and Rsk-1 inhibitor BI-D1870 was a product of BioVision Technologies. PKC ζ pseudosubstrate was purchased from Millipore, and Pin1 inhibitor AG-17724 was obtained from Sigma. Serum-starved MEF cells were pre-incubated with the inhibitor for 1 h prior to AGEs stimulation.

### Reconstitution of RelA-null MEFs with WT RelA and mutants

RelA mutants were generated with QuikChange II kit (Agilent Technologies), using wild-type RelA in pCDNA3.1(zeo+) vector (Invitrogen) as a template and primers carrying specific mutations. RelA (K218R/K221R/K310R) plasmid was a gift from Dr. Warner Greene (Gladstone Institute of Virology and Immunology, Addgene #2325), and the RelA (KR) insert from the original plasmid was subcloned to pCDNA 3.1 (zeo+). RelA-null MEFs were transfected with the plasmids using ViaFect transfection reagent (Promega) according to the manufacturer’s instruction, and the transfected cells were cultured in DMEM supplemented with 10% FBS and 400 μg/ml zeocin (Invitrogen). After 2 weeks, zeocin-resistant colonies were selected and grown in the same medium supplemented with 250 μg/ml zeocin. The expression of RelA wild-type and mutants in reconstituted RelA-null MEFs were confirmed with western blotting using antibodies to RelA (Santa Cruz), and cell lines that expressed a similar level of RelA as that of WT MEFs were selected for the studies.

### qRT-PCR analysis of *col1a1* and *col1a2* transcription

Total RNA from cells was isolated using TRI reagent (Sigma) according to the manufacturer’s instruction. SuperScript® III first-strand synthesis kit (Life Technologies) was used for reverse transcription. qRT-PCR for *col1a1* and *col1a2* genes were performed using QuantiFast SYBR green PCR kit (Qiagen) and analyzed on a 7300 Real Time PCR System (Applied Biosystems). Each reaction was performed in triplicate and all experiments were repeated 3 times. The transcripts of *col1a1* and *col1a2* were normalized with the housekeeping gene *Rn18S* and the relative mRNA expression levels were calculated according to the comparative 2^−∆∆CT^ method^61^. The primers used in the studies: *col1a1* sense: 5′-TGCTGGCAAAGATGGAGAAG-3′, *col1a1* antisense: 5′-CGGCAGGACCAGGAAGACC-3′; *col1a2* sense: 5′-CCGAGGCAGAGATGGTGTT-3′, *col1a2* antisense: 5′-GCAGCAAAGTTCCCAGTAAGA-3′; *Rn18S* sense: 5′-AACCCGTTGAACCCCATT-3′, *Rn18S* antisense: 5′- GGGCAGGGACTTAATCAACG-3′.

### Western blotting and antibodies used in the study

Approximately 0.5 × 10^6^ MEF cells were seeded in 35 mm plate one day before AGEs and/or inhibitor treatment. Cell extracts (10 μg of total protein) were resolved with SDS 4–12% precast gel (Life Technologies). Anti-RelA-, anti-Rsk-1, and anti-β–actin antibodies were from Santa Cruz Biotechnologies; antibodies to ERK1/2 and phosphorylated ERK1/2, phosphorylated Rsk-1(S380), and phosphorylated RelA S536 were from Cell Signaling Technology; antibodies to phosphorylated RelA S311 and RelA T254 were purchased from Abcam.

### ELISA analyses of AGEs-induced collagen I production

Murine VMSCs (0.5 × 10^6^) and macrophages (2 × 10^6^) were serum-starved, and treated with 50 μg/ml AGEs overnight. Cells were then harvested and lysed with 200 μl ELB buffer (50 mM Tris, pH 7.5, 300 mM NaCl, 0.1% Nonidet-P40, 5 mM EDTA, 1 mM phenylmethylsulfonyl fluoride, and 1 mM dithiothreitol). Lysates (10 μl) were diluted to 100 μl with ELB buffer and ELISA was performed using a collagen I assay kit (Biomatik) according to the manufacturer’s instruction.

### Chromatin immunoprecipitation (ChIP) assays

The ChIP assays were performed using Simple ChIP Plus Enzymatic Chromatin IP Kit (Cell Signaling Technology) according to the manufacturer’s instruction. Briefly, for each ChIP assay, 4 × 10^6^ MEF cells were used. Following cell lysis, micrococccal nuclease treatment and sonication, the obtained lysates were pre-cleared with anti-mouse IgG antibodies. After setting aside 2% lysates as input samples, the remaining lysates were incubated with anti-RelA antibodies (Cell Signaling Technology, ChIP grade) overnight at 4 °C with rotation. The lysates were then incubated with 30 μl Protein G agarose beads (ChIP grade) for 2 h at 4 °C with rotation. The beads were pelleted and washed 3 times with 1 ml low salt buffer, and incubated with 1 ml high salt buffer for 5 min at 4 °C with rotation. After centrifugation to remove the wash buffer, the ChIP samples were eluted with the elution buffer from the kit for 30 min at 65 °C. The eluted samples were then de-crosslinked, and purified with a spin column. Immunoprecipitated DNA and input DNA were analyzed by qPCR, and results were presented as a percentage of the input DNA. The primers used for ChIP assays are listed in Table S1.

### Statistical analyses

Data were expressed as mean ± standard error of the mean (SEM). For comparisons between two experimental groups, unpaired Student’s *t*-test was used to analyze the data. For comparisons among three or more experimental groups, data were analyzed using multisample comparison one-way ANOVA with post hoc Bonferonni corrections. Both analyses were performed using GraphPad Prism 6 statistical program. A value of *p* < 0.05 was considered statistically significant.

## Additional Information

**How to cite this article**: Peng, Y. *et al.* AGE-RAGE signal generates a specific NF-κB RelA “barcode” that directs collagen I expression. *Sci. Rep.* **6**, 18822; doi: 10.1038/srep18822 (2016).

## Supplementary Material

Supplementary Information
