# Supplementary material for: AGE-RAGE signal generates a specific NF-κB RelA “barcode” that directs collagen I expression
Source: Sci Rep. 2016 Jan 5;6:18822. doi: 10.1038/srep18822 (PMC4700418; doi:10.1038/srep18822)
Supplement: Supplementary Information [file srep18822-s1.pdf]

AGE-RAGE signal generates a specific NF-κB RelA "barcode" that directs collagen I expression

Yunqian Peng*, Ji-Min Kim*, Hal-Sol Park, Annie Yang, Celia Islam, Edward G. Lakatta & Li Lin¶

[1]Laboratory of Cardiovascular Sciences, National Institute on Aging, National Institute of Health, Baltimore, MD 21224.

*These authors contribute equally to this work.

¶Correspondence and requests for material should be addressed to L. L. (linli@mail.nih.gov)

SUPPLEMENTAL INFORMATION

Additional Methods

Vessel section and H & E staining. Thoracic aortas isolated from mice were fixed in 4% paraformaldehyde and embedded in paraffin. The paraffin block was then sectioned to 5 μm cross-sections. Rhesus monkey abdominal aorta sections were obtained from Dr. Mingyi Wang, NIA, NIH. The aorta sections were routinely stained with hematoxylin and eosin (H & E). Digital images of stained sections were obtained from light microscopy (Zeiss Axiovert 200) using polarized filters.

Immunohistochemistry (IHC). A standard avidin–biotin complex method was used for immunohistochemistry. Briefly, vessel sections were deparaffinized with xylene and rehydrated through gradient ethanol immersion. Sections were then microwaved for 5 min in citric acid buffer (2 mM citric acid and 9 mM trisodium citrate dehydrate, pH 6.0) to retrieve antigens. After washing with 1 x PBS, the slides were treated with peroxidase blocking buffer for 10 min

to quench endogenous peroxidase activity and blocked with serum blocking solution (Invitrogen) at room temperature for 1 h following washing and incubation with primary antibodies. Rabbit polyclonal anti-RAGE (1:1,000dilution), rabbit polyclonal anti-AGE (1:10,000dilution) and rabbit polyclonal anti-collagen 1 antibodies (1:500) were from Abcam. The specimens were then incubated with biotinylated secondary antibodies (Invitrogen), washed with Tris-buffered saline containing 0.1 % Tween-20, and re-incubated with streptavidin-horseradish peroxidase (Invitrogen) for detection. The sections were then counterstained with hematoxylin, rinsed, dehydrated, and mounted with mounting medium. The staining was visualized with a diaminobenzidine liquid substrate system (Vector Laboratory), and digitally captured with Zeiss Axiovert 200 microscope. Same quantitative analyses described in last method section were used for the IHC specimens. One-way ANOVA and post hoc Turkey's test are used determine significant differences between groups. The analysis was performed using GraphPad Prism 6 statistical program. $P < 0.05$ was considered to be significant.

Supplemental figure legends

Figure S1 Aging-associated AGEs, RAGE and collagen I increase in murine aortas. Thoracic aortas isolated from young (8 – 9 weeks) and old (40 – 45 weeks) male mice (WT and RAGE-null) were examined for (A) RAGE expression; (B) AGEs accumulation; (C) collagen I expression; (D) general histomorphology (H & E). The left panels are the representative staining samples from young and old animals; the right panels are the overall assessment. Four mice (n = 4) are in each group, and four randomly selected sections from each animal were analyzed. The data were presented as mean ± SEM, * $p < 0.05$; ** $p < 0.01$; *** $p < 0.001$.

Figure S2 Aging-associated AGE, RAGE and collagen I increase in rhesus monkey aortas. Abdominal aortas isolated from young (8 – 12 years) and old (23 – 25 years) rhesus monkeys

were examined for (A) RAGE expression; (B) AGEs accumulation; (C) collagen I expression. The left panels are representative aorta staining samples from young and old animals, the right panels are the overall assessment. Aortas from four young, and old monkeys (n = 4), and four randomly selected sections from each animal were analyzed. The data were presented as mean ± SEM, * $p < 0.05$.

Figure S3 AGEs stimulation does not induce RelA acetylation. (A) Immunoblotting of RelA acetyl lysine in anti-RelA antibodies precipitated AGEs-stimulated MEFs; (B) qRT-PCR analyses of transcription of *col1a1* and *col1a2* genes in AGEs-induced MEFs reconstituted with RelA (K218R/K221R/K310R). The data were presented as mean ± SEM, * $p < 0.05$. (C) Western blot using ant-RelA antibodies confirms the expression of RelA (K218R/K221R/K310R) and controls in MEFs.

Figure S4 AGEs stimulation induces phosphorylation of RelA S311 and T254 residues. MEF cells were treated with AGEs (50 μg/ml) and harvested at time points indicated. The cell lysates were resolved in SDS 4–12% gel and immunoblotted with anti-RelA, anti-p-RelA-T254, and anti-p-RelA-S311 antibodies (Abcam). (A) Time point of AGEs-induced RelA T254 and S311 phosphorylation, representative western blot; (B) and (C) Densitometric analyses of three western blots of (A); (D) and (E) pharmacological inhibitors treatment of MEFs induced with AGEs. Cells were pre-treated with the indicated inhibitor. After 1 h AGEs induction, the obtained cell lysates (10 μg of total protein) were resolved with SDS 4-12% precast gel and with anti-RelA, anti-p-RelA-T254 (D), and anti-p-RelA-S311 (E) antibodies. Anti- β actin antibodies were used as a loading control.

Supplemental Table S1

Primers used in ChIP assays.

Gene	Forward primer	Reverse primer	Locus
col1a1	GGGCAAAGATCCTGAGTTCC	AATCCTCTTGCCCAGTCTCC	-8618
col1a1	GGGCTAGGTTTCACAAAGGA	TGGGGAGTATTGCTCTCAAG	-7831
col1a1	CCCACCATGCTCCCTTCC	GCAGCAGATGTGGGAGAGAC	-7082
col1a1	CCGAGAGGCAGGGTTCCT	GGGGTTAGCTTCGGCTCA	+4
col1a2	TCCAAGCTGCAAAAATAAATTT	CAACTGTGGAGGTCTTATTGTG	-9355
col1a2	TTTGGATCTTTTGGGTGCT	GAGCCCAGGTGACTCATTG	-8034
col1a2	GAGTTCTGGGGAGTTAGCC	CATCAGCAATCATTAACTAAATC	-5906
col1a2	TGTAAGTCAGAGAGGGCATCTA	CATAACTCAGAGGGATGTGC	-5386

Figure S1

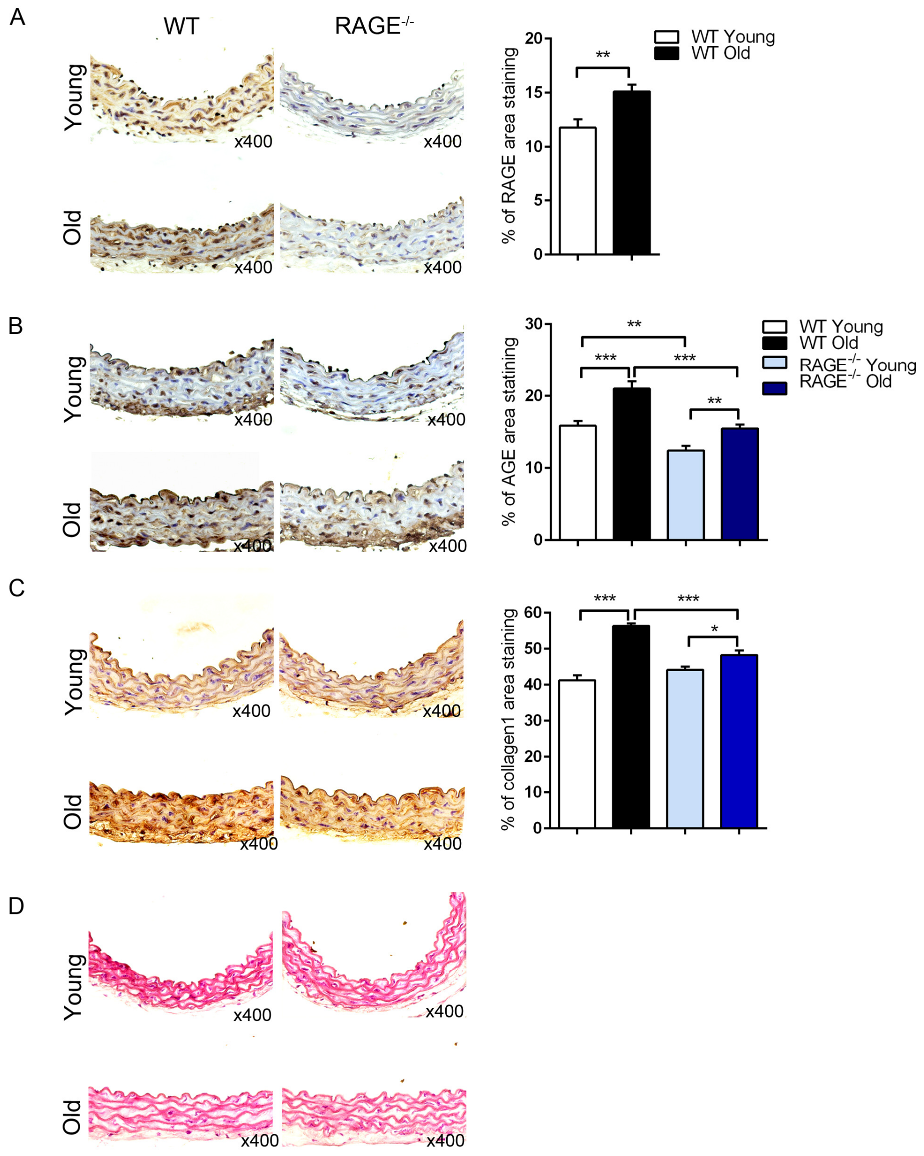

Figure S2

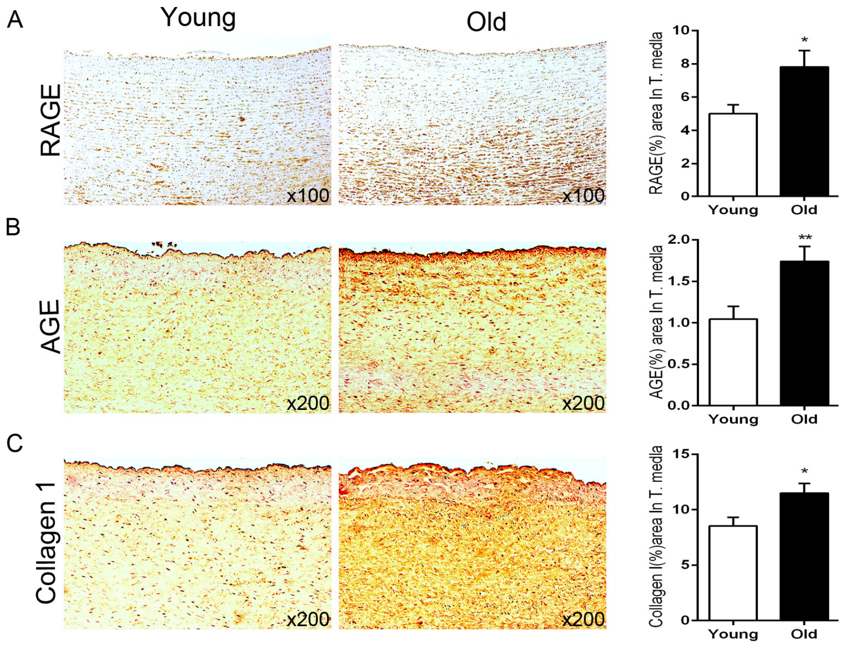

Figure S3

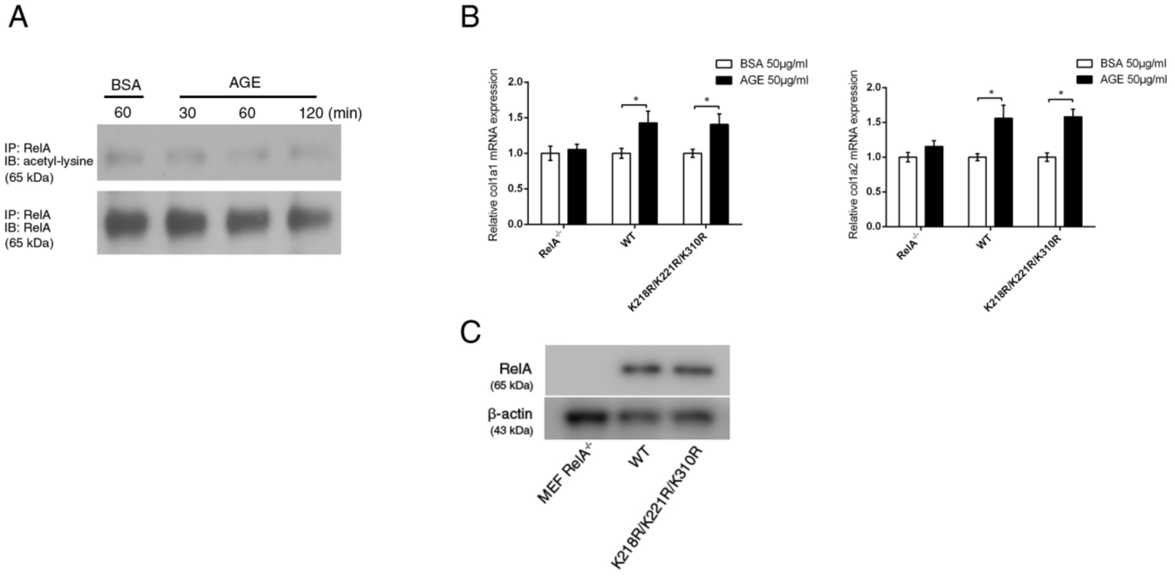

Figure S4

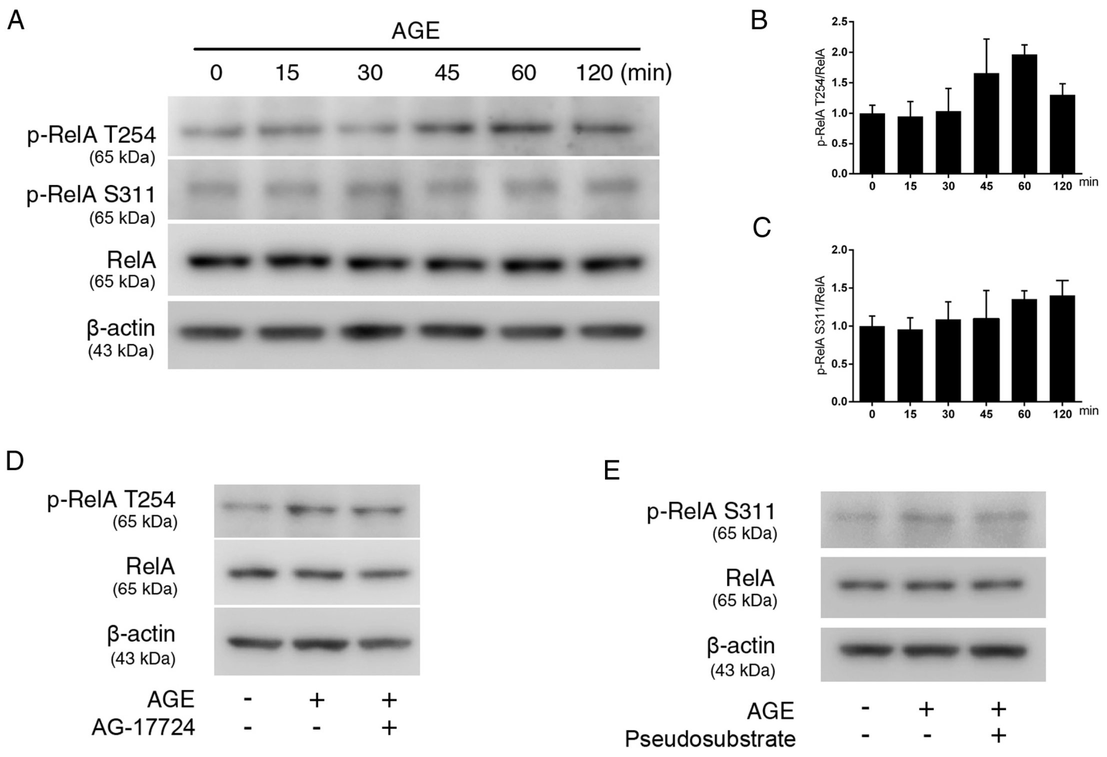

A
AGE
0
15
30
45
60
120 (min)
p-RelA T254
(65 kDa)
p-RelA S311
(65 kDa)
RelA
(65 kDa)
β-actin
(43 kDa)
B
p-RelA T254/RelA
min
C
p-RelA S311/RelA
min
D
p-RelA T254
(65 kDa)
RelA
(65 kDa)
β-actin
(43 kDa)
AGE - + +
AG-17724 - - +
E
p-RelA S311
(65 kDa)
RelA
(65 kDa)
β-actin
(43 kDa)
AGE - + +
Pseudosubstrate - - +
